# On measuring and decomposing inequality of opportunity in access to health services among Tunisian children: a new approach for public policy

**DOI:** 10.1186/s12955-017-0777-7

**Published:** 2017-10-25

**Authors:** Anis Saidi, Mekki Hamdaoui

**Affiliations:** 1 Faculty of Economic Sciences and Management of Sousse (FSEGSousse), Sousse, Tunisia; 2 Faculty of Economics Sciences and Management of Tunis (FSEGT), Tunis, Tunisia

**Keywords:** Inequality of opportunity, Dissimilarity index, Tunisia, Children, D63, D30

## Abstract

**Background:**

The early years in children’s life are the key to physical, cognitive-language, and, socio-emotional skills development. So, it is of paramount importance in this period to be interested in different indicators that would influence the child’s health.

**Methods:**

This paper measures inequality of opportunities among Tunisian children concerning access to nutritional and healthy services using Human Opportunity-Index and Shapely decomposition methods.

**Results:**

Many disparities between regions have been detected since 1982 until 2012. Tunisian children face unequal opportunities to develop in terms of health, nutrition, cognitive, social, and emotional development. Likewise, we found that, parents’ education, wealth, age of household head and geographic factors as key factors determining child development outcomes.

**Conclusion:**

Our findings suggested that childhood unequal opportunities in Tunisia are explained by pension funds deficiency and structural problem in the labor market.

**Trial registration:**

The results of a health care intervention on human participants “retrospectively registered”.

## Background

World Development Organizations seek to reduce the proportion of people who suffer from hunger. A reduction in the prevalence of malnutrition can contribute to the reduction of infant mortality. However, countries tend to under-invest in this stage of development, particularly in developing countries. Inequality of opportunity in early childhood is studied across the early life course and is often quantified until age five in terms of health, nutrition, social-emotional development, early learning, and early work and explained by many circumstances such us access to health services.

Likewise, a reduced regional disparity is an important determinant of long run growth and development and contributes to guarantee political and economical stability. Furthermore, variation in disease environments could contribute to inequality in health outcomes related to place of residence [1].

Despite the importance of early childhood, there is limited research on the state of early childhood development and inequality in Tunisia. This issue is frequently absent from political agendas, insufficiently researched, and under-resourced. In this paper, we examine the inequality of opportunity that children in Tunisia face in early childhood across a variety of basic services access and decompose inequality of opportunity in order to identify its determinants. This analysis not only contributes to the improvement of limited research on early children development and inequality in Tunisia, but also provides critical information for identifying the vulnerable groups, key issues, and factors that limit children’s development early in life. Our contribution is to take into consideration multidimensional aspects of inequality to overcome shortcomings linked to previous one-dimensional methodology.

Equality of opportunity is based on the distinction between efforts and circumstances that are under and beyond the individual’s control [1, 2]. So unequal opportunities result from a big difference in circumstances such as: family background sex, place of birth… the ways of dealing with such circumstances have being unfair and require quick and efficient action from political decision makers. Constraints on access to services and basis resources contribute to perpetuate the lack of both capacities and opportunities in a large part of society [3, 2, 4].

The early years in the child’s life cycle are considered as the fundamental starting point of inequality of opportunity at the physical cognitive and especially psychological level bearing in mind that these competencies develop early in life [5]. In other way, well-brought up and well surrounded children have better chances to develop their knowledge [6], communication, social competencies, and grow healthy while having high self-esteem [7, 8]. The early years of life have been described by some people as “a prolonged critical period and a real window opportunity for development that ends at three years stage” [9].

Underfeeding has a negative impact on economic and social development. Its effect can persist up to advanced stages in a human being’s life and particularly children [9]. Throughout research, a number of studies show that biological and psycho-social risks affect individual development considerably by means of changes in structure and function of the brain which can lead to behavior changes, the latter will doubtlessly lead to a significant impact on the life of the individual and society [10].

To assess the extent of inequality in early childhood, we draw on the concepts and methodology developed in the recent literature on inequality of opportunity (De [11, 12, 2, 13]). Using data from a surveys covering Tunisia, we examine the state of early childhood development in terms of early health services. We quantify the unequal opportunities children have to develop along health services using the dissimilarity index (De [11]) and decompose inequality into the contributions of different circumstances using the Shapley decomposition [13].

Inequality of opportunity in Tunisia is particularly high in access to health services between regions and in activities that support early cognitive development, which has important implications for inequality in children’s subsequent labor force. Our analysis also illustrates the pathways through which circumstances shape children’s early opportunities. Overall, wealth, mother’s education, and geographic differences tend to contribute substantially to inequality of opportunity. This paper is the first paper that measures inequality of opportunities among children in Tunisia on selected health utilization, nutrition indicators using the Human Opportunity Index (HOI), which is a measure of inequality of opportunity in basic services for children.

Before presenting our findings in section 4, we organized our paper us follow: In section 2, we present a conceptual framework for inequality of opportunity in early childhood development. Section 3 describes our empirical strategy and discusses the surveys and samples. Finally, section 5 provides implications of our findings and conclusions.

## A conceptual framework

Based on the philosophical works elaborated by Rawls [14], Sen. [15], Dworkin [16, 17], Cohen [18]; Arenson [19] and Roemer [20, 2], was the first to have introduced the concept of equality of chances in the economic literature. They distinguished between effort and circumstances in explaining divergences in wealth an opportunity in adulthood. The circumstances are defined as factors on which individuals have no control such as: ethnical origin sex, age, parental education…etc. This inequality of chances is widely considered unfair and deserving of attention from policy makers.

Our approach in this paper is based on Roemer’s frameworks (1998) who present “model of advantage” to decompose outcomes into a controllable part (effort) and a non controllable condition(circumstances) that the States must intervene to reduce in order to guaranty social equity. This model can be presented as follow:

1 $$ y=f\left(C,E,u\right) $$

Where *y*, designates the considered outcome, *C* and *E* are respectively vectors of circumstances and effort variables and *u* represents the random factors. As noted above, Roemer’s theory (1998) presumes explicitly that circumstances must be economically exogenous i.e. the person can’t control over them. Conversely, efforts may be endogenous and may therefore depend on circumstances as shown in the following equation:

2 $$ y=f\left[C,E\left(c,v\right),u\right] $$

According to Roemer, realizing an equality of opportunities requires that F(y/C) = F(y) which means simultaneously that no circumstance variable should have a direct causal impact on variable y (∂f(C, E, u)/ ∂ C = 0), each effort variable should be distributed independently from all circumstances G(y/C) = G(y). Furthermore, Random factors are independent from circumstances H(y/C) = H(y) where all three functions F, G and H denote cumulative distributions. Subsequently, an inequality of opportunity occurred when F(y/C) ≠ F(y) and the extent of this inequality could be measured by the difference between the two members of the previous inequality. This last inequality has been defined as Roemer’s strong definition of inequality of opportunity in a several recent papers, including Bourguignon et al., [3]; Ferreira and Gignoux [21].

So, earlier literature seeking to separate the effect of efforts from circumstances (out of control) has led to the emergence of the concept “Human opportunity index”. It corresponds to a synthetic measure of opportunities inequality, proposed for the first time by the social welfare function of Sen [22] and developed by the Word Bank on 2006. This index is firstly applied to measure inequality of opportunity in access to basic services in Latin America and Caraib by De Barro and al., [23]. Since then, this measure has been widely used in the literature of inequalities but the results are different may be because of the used measures of inequalities. This tool has the advantage of giving an idea on the level of accessibility to any service by a given population and gives the level of discrepancies in sample in terms of access to this service. In other words, it helps respond to these preoccupations: (i) How many opportunities are available to a childhood in any region of a given country (the coverage rate by a basic service). (ii) How equitably those opportunities are distributed (whether the dissimilarity in individual access to the same service is due to exogenous circumstances and inequality of chances). We are largely based on the idea presented in this section in developing our methodology. We constructed a conceptual and empirical frameworks permitting us explain inequality in access to basic services by Tunisian children.

## Data and methodology

### Data choice and descriptions

We use data from the Multiple Indicator Cluster Surveys (MICS4), this survey was executed in 2011-2012 by the Ministry of Development and Cooperation with the National Institute of Statistics of Tunisia (INS), financial and technical support was provided by the United Nations Children’s Emergency Fund (UNICEF), the United Nations Population Fund (UNFPA) and the Swiss Cooperation Office in Tunisia. It is the only recent database available until our day, which contains rich information on the situation of women and children in this country.

We use also data concerning place of residence, socio-economic and demographic indicators for three governorates of the center (Kasserine, Kairouan and Zidi-Bouzid) and for six regions of the country (District Tunis, North East, North West, Center East, South East and South West). Otherwise, we use 8 variables of circumstances: residence, age of household’s head, family wealth index, sex of household head, gender, number of children per household, level of education of household head and household size.

Firstly, to study nutrition situation of Tunisian children we are based on a sample of 9600 selected households where 2938 children under 5 years were identified through the household question sheet. This question sheet was filled for 2768 of these children, which corresponds to a 94.2% answer rate among households with children under 5 years interviewed [24]. Descriptive statistics containing demographic information about of this sample are presented in the Table 10 Appendix. Then, to analyze the development of babies’ health in Tunisia, we use crucial index measuring opportunity access to basic services using data provided by the INS (2011-2012). The database covers 9867 women interviewed, of whom 4204 gave birth and 1059 gave birth during the last 2 years before the interview. The first sample of women, that have had children since 1982 until 2012, allows us to see the disparities in terms of access to basic health services for children. The last database which contains 1059 women who gave birth in the last years preceding the questionnaire is important in the sense that it allows us to follow the evolution of inequalities of chances in relation to previous years.

For the choice of our variables, we are based on important indicators and outcomes identified in previous literature, and as constrained by the data availability, we considered nutritional and health care utilization variables as our proxy for health services access.

The nutritional status of children is a reflection of their overall health. When children have access to adequate food, are not exposed to repeated morbid episodes and are healthy, they reach their growth potential and are considered well fed. Malnutrition is responsible for more than half of all child deaths worldwide. Undernourished children are more likely to die from common childhood illnesses and those who survive have recurrent diseases and stunted growth. One of the main goals of World Health Organization is to reduce the proportion of people who suffer from hunger. A reduction in the prevalence of malnutrition will also help to reduce infant mortality. In a well-nourished population, there is a reference distribution of the size and weight of children under 5 years of age. Under-nutrition in a population can be measured by comparing children to the reference population. The reference population used in this work is based on the WHO growth standards. Each of the three indicators of nutritional status can be expressed in units of standard deviations (reduced deviation) from the median of the reference population (Tables 13 and 14 in the Appendix).

**Weight-for-age** is a measure of both acute and chronic malnutrition. Children whose weight-for-age is more than two standard deviations below the median of the reference population are considered to be low or moderate underweight, while those whose weight-for-age is more than three standard deviations below the median are considered to be severely underweight(Table 13).

**The length-for-age** is a measure of linear growth. Children whose height-for-age is more than two standard deviations below the median of the reference population are considered to be too small for their age and are classified as having moderate or severe growth retardation. Those whose height-for-age is more than three standard deviations below the median are classified as having severe growth retardation. Stunting is a reflection of chronic malnutrition resulting from lack of adequate nutrition over a long period of time and from recurrent or chronic diseases (Table 13).

Finally, children whose **weight-for-height** is more than two standard deviations below the median of the reference population are classified as moderately or severely emaciated, while those with more than three standard deviations below the median are considered severely emaciated. Emaciation is generally the result of a recent nutritional deficiency. The indicator may have significant seasonal variations associated with changes in food availability or disease prevalence (Table 14).

Table 1 shows the percentages of children in each of these categories, based on the anthropometric measurements taken during the fieldwork. Based on the new WHO growth standards,^1^ 2.57% of children under 5 years old in Tunisia are underweight (moderate or severe). Approximately one of ten children (10.33%) suffers from moderate or severe stunting and 2.2% are moderately or severely emaciated.

**Table 1:** Basic characteristics of children under 5 years according to selected characteristics (Nutrition)

Tunisia (2011-2012)			Nutrition: Weight for Age		Nutrition: Height for Age		Nutrition: Weight for height	
			Underweight	No ponderal insufficiency	Growth delay	No growth delay	Emarciation	No emarciation
	Total	2768 100.00	71 2.57	2697 97.43	286 10.33	2482 89.67	61 2.20	2707 97.80
Gender	Male	1482 53.54	48 3.24	1434 96.76	163 11.00	1319 89.00	40 2.70	1442 97.30
	Female	1286 46.46	23 1.79	1263 98.21	123 9.56	1163 90.44	21 1.63	1265 98.37
Residence	Urbain	1607 58.06	41 2.55	1566 97.45	126 7.84	1481 92.16	38 2.36	1569 97.64
	Rural	1161 41.94	30 2.58	1131 97.42	160 13.78	1001 86.22	23 1.98	1138 98.02
Region	District Tunis	356 12.86	5 1.40	351 98.60	24 6.74	332 93.26	10 2.81	346 97.19
	North East	379 13.69	8 2.11	371 97.89	37 9.76	342 90.24	6 1.58	373 98.42
	North west	291 10.51	10 3.44	281 96.56	38 13.06	253 86.94	4 1.37	287 98.63
	Centre East	308 11.13	5 1.62	303 98.38	18 5.84	290 94.16	8 2.60	300 97.40
	Kasserine	282 10.19	5 1.77	277 98.23	39 13.83	243 86.17	7 .48	275 97.52
	Kairouan	305 11.02	11 3.61	294 96.39	40 13.11	265 86.89	5 1.64	300 98.36
	Sidi Bouzid	250 9.03	10 4.00	240 96.00	33 13.20	217 86.80	6 2.40	244 97.60
	South East	347 12.54	6 1.73	341 98.27	24 6.92	323 93.08	9 2.59	338 97.41
	South Ouest	250 9.03	11 4.40	239 95.60	33 13.20	217 86.80	6 2.40	244 97.60
Mather’s education	Nothingness	466 16.84	21 4.51	445 95.49	79 16.95	387 83.05	9 1.93	457 98.07
	Primary and similar	917 33.13	16 1.74	901 98.26	101 11.01	816 88.99	17 1.85	900 98.15
	Secondary and similar	951 34.36	23 2.42	928 97.58	79 8.31	872 91.69	21 2.21	930 97.79
	Superior	434 15.68	11 2.53	423 97.47	27 6.22	407 93.78	14 3.23	420 96.77
Annual family incomes (Economic quintile)	The poorest	737 26.63	30 4.07	707 95.93	118 16.01	619 83.99	13 1.76	724 98.24
	Second	606 21.89	11 1.82	595 98.18	72 11.88	534 88.12	14 2.31	592 97.69
	Medium	479 17.30	11 2.30	468 97.70	29 6.05	450 93.95	9 1.88	470 98.12
	Fourth	565 20.41	11 1.95	554 98.05	46 8.14	519 91.86	13 2.30	552 97.70
	The richest	381 13.76	8 2.10	373 97.90	21 5.51	360 94.49	12 3.15	369 96.85

The second value in the table corresponds to the percentage contribution in the corresponding sample

There are also variations in anthropometric indicators according to socio-demographic characteristics; boys appear to be slightly more likely than girls to accuse underweight, stunting, and emaciation. Disparities by environment are characterized by a higher prevalence of moderate or severe growth retardation in rural areas (≈14%) than in urban areas (8%). In terms of geographical variations, we can see a higher prevalence of underweight in the South West, Sidi Bouzid, Kairouan and North West (4%), while the prevalence of moderate or severe growth problem is touched in Kasserine (13.83%), in south-west, sidi bouzid, kairouan and north-west (more than 13%).

Children whose mothers/guardians with secondary or superior education are the least likely to be underweight and stunted compared to the children of mothers who have never attended school. As for the disparities according to the level of economic well-being, the prevalence of underweight and stunting are higher among the poorest.

Similarly, the prenatal period offers important opportunities to provide services that may be essential to the health of pregnant women and their infants [25]. A better understanding of the growth and development of the fetus and its relationship to maternal health has led to increased attention to prenatal care, which has been widely demonstrated to have an impact on improving maternal and neonatal health. For example, if the prenatal period is used to inform women and families about warning signs, symptoms and risks related to labor and delivery, it can guide women to give birth in the best possible way with the assistance of qualified care personnel. The prenatal period also provides an opportunity to provide information on birth spacing, recognized as an important factor in improving infant survival. Tetanus vaccination during pregnancy can save both mother and infant life. Preventing and treating malaria in pregnant women, managing anemia during pregnancy and treating STIs (sexually transmitted infections) can greatly improve the chances of survival of the fetus and the health of the mother. Adverse outcomes such as low birth weight can be prevented through a combination of interventions to improve the nutritional status of women and prevent infections (eg, malaria and STIs) during pregnancy. More recently, the potential of the prenatal period as an entry point for the prevention of HIV (Human Immunodeficiency Virus) and care, especially for the prevention of mother-to-child transmission of HIV, has lead to renewed interest in the access and use of prenatal care services.

World Health Organization recommends a minimum of four antenatal visits based on an analysis of the effectiveness of different antenatal care models. WHO guidelines are specific to the content of prenatal consultations, including: measurement of blood pressure; Urine analysis for bacteriuria and proteinuria; Blood testing to detect syphilis and severe anemia; and weight/length measurement (optional).

In this framework, we present the level of health care coverage in Table 2 and the type of staff providing prenatal care to women aged 15-49 who gave birth in the two years preceding the survey in Table 15 Appendix. This table shows that access to antenatal care is relatively high in the country as a whole with 97.83% of women receiving prenatal care at least one time during pregnancy (79.03% per doctor and 44.47% per auxiliary midwife). The highest levels of prenatal care are observed in the South East and South West regions (100%); while the lowest level is in the Sidi Bouzid region (89.36%). There are few differences among children following residence (98.50% in urban areas versus 96.94% in rural areas). This coverage is around 97.06% for boys and 98.64% for girls. It increases with women’s educational attainment (from 95.55 to 100%) and the level of economic well-being of households. Of the women surveyed and concerned with antenatal care, 79.03% were examined by a physician during pregnancy; this proportion is higher in urban areas (82.69%) than in rural areas (74.23%). It is higher among women residing in the Central East region (93.57%), women with university education (93.10%), and women in the richest household category (97.87%). The lowest proportions were found among women who had never attended school (67.04%) and those in the governorate of Kairouan (67.50%) and the South West region (68.57%). This level of coverage has been low in previous decades and is approaching an average of 25% throughout the study period. The distribution is similar for blood samples with a slight decrease in the level of coverage, which drops to 94.62% in 2012 and does not exceed 24% (23.86%) over the period from 1982 until the date of the survey always with a small advantage of the southern regions.

**Table 2:** Basic characteristics of children under 5 years according to selected characteristics (Health)

Tunisia		Tunisia 1982-2012							Tunisia 2011-2012						
			Health: Prenatal care		Health: Blood sample		Health: Post natal care			Health: Prenatal care		Health: Blood sample		Health: Post natal care	
		Total	No access	acces	No access	access	No access	acces	Total	No access	acces	No access	acces	No access	acces
		4200 100.00	3164 75.33	1036 24.67	3198 76.14	1002 23.86	3598 85.67	602 14.33	1059 100.00	23 2.17	1036 97.83	57 5.38	1002 94.62	457 43.15	602 56.85
Gender	Male	2084 49.62	1555 74.62	529 25.38	1568 75.24	516 24.76	1782 85.51	302 14.49	545 51.46	16 2.94	529 97.06	29 5.32	516 94.68	243 44.59	302 55.41
	Female	2116 50.38	1609 76.04	507 23.96	1630 77.03	486 22.97	1816 85.82	300 14.18	514 48.54	7 1.36	507 98.64	28 5.45	486 94.55	214 41.63	300 58.37
Residence	Urbain	2613 62.21	2021 77.34	592 22.66	2036 77.92	577 22.08	2264 86.64	349 13.36	601 56.75	9 1.50	592 98.50	24 3.99	577 96.01	252 41.93	349 58.07
	Rural	1587 37.79	1143 72.02	444 27.98	1162 73.22	425 26.78	1334 84.06	253 15.94	458 43.25	14 3.06	444 96.94	33 7.21	425 92.79	205 44.76	253 55.24
Region	District Tunis	629 14.98	497 79.01	132 20.99	499 79.33	130 20.67	551 87.60	78 12.40	135 12.75	3 2.22	132 97.78	5 3.70	130 96.30	57 42.22	78 57.78
	Nord Est	586 13.95	443 75.60	143 24.40	449 76.62	137 23.38	485 82.76	101 17.24	146 13.79	3 2.05	143 97.95	9 6.16	137 93.84	45 30.82	101 69.18
	Nord Ouest	516 12.29	405 78.49	111 21.51	410 79.46	106 20.54	452 87.60	64 12.40	112 10.58	1 0.89	111 99.11	6 5.36	106 94.64	48 42.86	64 57.14
	Centre Est	478 11.38	370 77.41	108 22.59	375 78.45	103 21.55	391 81.80	87 18.20	109 10.29	1 0.92	108 99.08	6 5.50	103 94.50	22 20.18	87 79.82
	Kasserine	393 9.36	294 74.81	99 25.19	299 76.08	94 23.92	328 83.46	65 16.54	102 9.63	3 2.94	99 97.06	8 7.84	94 92.16	37 36.27	65 63.73
	Kairouan	365 8.69	247 67.67	118 32.33	249 68.22	116 31.78	306 83.84	59 16.16	120 11.33	2 1.67	118 98.33	4 3.33	116 96.67	61 50.83	59 49.17
	Sidi Bouzid	348 8.29	264 75.86	84 24.14	269 77.30	79 22.70	307 88.22	41 11.78	94 8.88	10 10.64	84 89.36	15 15.96	79 84.04	53 56.38	41 43.62
	Sud Est	472 11.24	336 71.19	136 28.81	337 71.40	135 28.60	415 87.92	57 12.08	136 12.84	0 0.000	136,100.00	1 0.74	135 99.26	79 58.09	57 41.91
	Sud Ouest	413 9.83	308 74.58	105 25.42	311 75.30	102 24.70	363 87.89	50 12.11	105 9.92	0 0.000	105 100.00	3 2.86	102 97.14	55 52.38	50 47.62
Mather’s education	nothingness	405 22.64	321 79.25	84 20.75	325 80.24	80 19.76	354 87.40	51 12.60	88 17.46	4 4.45	84 95.55	8 9.09	80 90.91	37 42.04	51 57.96
	Primary and similar	645 36.05	476 73.80	169 26.20	487 75.50	158 24.50	558 86.51	87 13.49	172 34.13	3 1.74	169 98.26	14 8.14	158 91.86	85 49.42	87 50.58
	Secondary and similar	538 30.07	385 71.56	153 28.44	388 72.12	150 27.88	448 83.27	90 16.73	157 31.15	4 2.55	153 97.45	7 4.46	150 95.54	67 42.68	90 57.32
	Superior	201 11.24	114 56.72	87 43.28	115 57.21	86 42.79	148 73.63	53 26.37	87 17.26	0 0.00	87 100.00	1 1.15	86 98.85	34 39.08	53 60.92
	No reponse	2411 57.40	1868 77.48	543 22.52	1883 78.10	528 21.90	2090 86.69	321 13.31	555 52.40	12 2.16	543 97.84	27 4.86	528 95.14	234 42.16	321 57.84
Annual family incomes(Economic quintile)	The poorest	1047 24.93	774 73.93	273 26.07	785 74.98	262 25.02	904 86.34	143 13.66	288 27.20	15 5.21	273 94.79	26 9.03	262 90.97	145 50.35	143 49.65
	second	850 20.24	622 73.18	228 26.82	632 74.35	218 25.65	720 84.71	130 15.29	230 21.72	2 0.87	228 99.13	12 5.22	218 94.78	100 43.48	130 56.52
	medium	774 18.43	600 77.52	174 22.48	608 78.55	166 21.45	680 87.86	94 12.14	179 16.90	5 2.79	174 97.21	13 7.26	166 92.74	85 47.49	94 52.51
	fourth	791 18.83	571 72.19	220 27.81	574 72.57	217 27.43	659 83.31	132 16.69	221 20.87	1 0.45	220 99.55	4 1.81	217 98.19	89 40.27	132 59.73
	the richest	738 17.57	597 80.89	141 19.11	599 81.17	139 18.83	635 86.04	103 13.96	141 13.31	0 0.00	141 100.00	2 1.42	139 98.58	38 26.95	103 73.05

The second value in the table corresponds to the percentage contribution in the corresponding sample

In Tunisia, two postnatal consultations are recommended: on the eighth and fortieth day after childbirth [26].. However, no question on these two visits is included in the questionnaire. This survey revealed that 85.67% of newborns had no postnatal consultation during the first 6 days after birth between 1982 and 2012, while 43.15% born in the 2 years prior to the survey received no postnatal care (Table 2). This percentage is the highest in Sidi Bouzid (88.22% over the entire period and 56.38% in 2012) and it is the lowest in the Center East (81.80 and 20.18%). There are few differences on average between urban areas (86.64%) and rural areas (84.06%). This percentage decreases with the level of economic well-being and with the level of schooling of the mother.

### Methodology

As indicated previously, we aim to study inequality in early childhood access to basic services. Otherwise, our variables of interest are binary meaning two possibilities either access or not. So, we follow De Barros [23], Son [27] to define a dichotomous variable *zi* which takes a value of 1 if the *i*th person of specific group has access to basic opportunity and takes a value of 0 if he lacks access to the considered opportunity. It can be readily proved that (*zi*) = *pi* = (*zi*), where *pi* is the average accomplishment related to the dichotomous outcome (zi) with respect to a specific group of sample. *pi* could be defined otherwise as the probability that the *i*th person has access to a given opportunity. It depends on a vector of exogenous variables indicating the socioeconomic circumstances (such as gender, age, area of residence…) of each group, the total characteristic being *k*. There can be as many probability gaps between individuals/groups as there are possible combinations of group-identifying circumstances (income groups, household-size groups, gender groups…).

Given a set of *k* circumstance variables *xi*1, *xi*2… *xi*k, we estimate the probability *pi* for each child (In this study we focus particularly on children as we assume that many of the differences in opportunities are generated during childhood and carried out the whole life) by means of a logit model. Accordingly, we have the following expression of:

3 $$ {\mathrm{p}}_{\mathrm{i}}=\frac{{\mathrm{e}}^{\left({\upbeta}_0+\sum \limits_{\mathrm{j}=1}^{\mathrm{k}}{\upbeta}_{\mathrm{j}}{\mathrm{x}}_{\mathrm{i}\mathrm{j}}\right)}}{1+{\mathrm{e}}^{\left({\upbeta}_0+{\sum}_{\mathrm{j}=1}^{\mathrm{k}}{\upbeta}_{\mathrm{j}}{\mathrm{x}}_{\mathrm{i}\mathrm{j}}\right)}} $$

Secondly, we compute the overall coverage rate $\overline{\mathrm{p}}$ which is the proportion of the population with access to a given opportunity using the following formula:

4 $$ \overline{\mathrm{p}}={\sum}_{\mathrm{i}=1}^{\mathrm{n}}{\mathrm{w}}_{\mathrm{i}}{\widehat{\mathrm{p}}}_{\mathrm{i}} $$

Where ${\mathrm{w}}_{\mathrm{i}}=\frac{1}{\mathrm{n}}$ and n is the size of sample considered. Then, the Dissimilarity Index $\widehat{\mathrm{D}}$ can be computed as follows:

5 $$ \widehat{\mathrm{D}}=\frac{1}{2\overline{\mathrm{p}}}{\sum}_{\mathrm{i}=1}^{\mathrm{k}}{\mathrm{w}}_{\mathrm{i}}\left|{\widehat{\mathrm{p}}}_{\mathrm{i}}-\overline{\mathrm{p}}\right| $$

After calculating the penalty which is equal to P = C × D, we get the final formula of the HOI for each service or outcome:

6 $$ \mathrm{HOI}=\overline{\mathrm{p}}\ \left(1-\mathrm{D}\right) $$

Human opportunity index specification provides an overview in the differences between regions in terms of percentage coverage by any service in addition to dissimilarity level but it is silent about origin of inequality. To overtake this limit, we refer to **Shapley Decomposition methodology that consists in identifying how each circumstance “contributes” to Inequality in access to basic services** [28, 29, 13].^2^ This approach extends the idea of the Shapley value of cooperative games into applications for decomposing inequality. The decomposition consists of calculating the marginal contributions of each circumstance as they are removed in sequence. Following Barros et al. [11], and [13], we can measure inequality of opportunities by the penalty (P) or by the dissimilarity index (D), as defined in expressions (4) and (5) above. The value of these two measures–where P is just a scalar transformation of D–is dependent on the set of circumstances considered. Moreover, they have the important property that adding more circumstances always increases the value of P and D. If we have two sets of circumstances A and B, and set A and B do not overlap, then HOI(A,B) ≤ HOI(A); and alternatively, D(A,B) ≥ D(A). The impact of adding a circumstance A is given by:

$$ \left[\mathrm{D}\right(\mathrm{s}\left\{\mathrm{A}\right\}-\mathrm{D}\left(\mathrm{S}\right)\Big] $$

7 $$ {\mathrm{D}}_{\mathrm{A}}={\sum}_{\mathrm{S}\subseteq \mathrm{N}\backslash \left\{\mathrm{A}\right\}}\frac{\left|\mathrm{s}\right|!\left(\mathrm{n}-\left|\mathrm{S}\right|-1\right)!}{\mathrm{n}!}\left[\mathrm{D}\left(\mathrm{S}\cup \left\{\mathrm{A}\right\}\right)-\mathrm{D}\left(\mathrm{S}\right)\right] $$

Where *N* is the set of all circumstances, which includes *n* circumstances in total; *S* is a subset of *N* that does not contain the particular circumstance *A*. *D(S)* is the dissimilarity index estimated with the set of circumstances *S*. D (S U{A}) is the dissimilarity index calculated with set of circumstances *S and* the circumstance *A*. The contribution of circumstance A to the dissimilarity index can be defined as:

8 $$ {\mathrm{M}}_{\mathrm{A}}=\frac{{\mathrm{D}}_{\mathrm{A}}}{\mathrm{D}\left(\mathrm{N}\right)}\ \mathrm{where}\ {\sum}_{\mathrm{i}\in \mathrm{N}}{\mathrm{M}}_{\mathrm{i}}=1 $$

We measure variations in HOI in Tunisia in the time period surveyed based on 2 main indicator categories: (i) Malnutrition Intake, and (ii) Healthcare utilization before, during pregnancy to healthcare services in early year using data from the 2011 and 2012 (MICS4) samples.

## Results and discussions

We present our results and interpretations in terms of coverage beginning by the nutritional status of children in Tunisia during the period of the survey elaboration then by access to health care services before, after, and during pregnancy.

### Access to nutritional services by Tunisian childhood

#### Results

Given the importance of nutrition and its influence on the health status and early childhood mortality rate, it should be noted that in a well-nourished population there is a standard distribution of the height and weight of children less than five years aged. Under-nutrition in a population can be measured by comparing children to the reference population.^3^ Stunting indicates accumulated malnutrition, damages psycho-social development [30] and engenders poorer school performance leading to lower productivity and so wages later in life, according to classical theory [31]. Indeed, it results that there are variations of the anthropometric indicators according to the socio-demographic characteristics.

Table 3 shows that for the first model, when we consider weight for age ratio as the dependent variable, household’s size increase significantly at the 5% threshold underweight problem.^4^ However, head’s household age, number of children (2-14) per household and head’s household education level decreases significantly the probability of children to suffer from problem of underweight. Concerning determinants of children’s stunting, it seems that household’s education level, high family income, male nature and age of head’s household significantly reduces the likelihood to have problem of growth during the first five years of birth (second column). Similarly, a child who belongs to a large family may significantly have problems of emaciation, whereas if he or she lives with more than one child (2-14) he or she becomes more protected against this type of problem (last column).

**Table 3:** Results of logit model (Nutrition)

Endogenous variables	Nutrition: Weight for Age		Nutrition: Height for Age		Nutrition: Weight for height	
Exogenous Variables	Coef	*P*-Value	Coef	*P*-Value	Coef	*P*-Value
Gender	−.041	0.865	−.033	0.794	−.315	0.233
Residence	−.488	0.103	.191	0.208	−.166	0.610
Head’s household Education	.865	0.022	.565	0.004	.418	0.368
Household income	.355	0.228	.605	0.000	−.110	0.727
Head’s household gender	−1.07	0.294	.747	0.003	−1.00	0.331
Household size	−.417	0.000	−.064	0.256	−.227	0.023
Number of children (2-14)	.288	0.011	.014	0.828	.415	0.002
Head’s household age	.063	0.000	.019	0.009	.024	0.131
Constant	3.13	0.010	.128	0.763	4.16	0.001
Obs	2768		2768		2768	
Prob > chi2	0.0000		0.0000		0.0543	

Table 4 presents results of HOI regressions which give an idea about nutritional status of children in each region in the Tunisian areas. If we interpret our results in terms of coverage, we can see that it is almost satisfactory for the 3 indicators of nutrition such us weight for age, height for age and weight for height are respectively 97.43%, 89.66%, and 97.79%.

**Table 4:** Rate of anthropometric indicators coverage by region

	Weight for age (Malnutrition %)	Height for age (stunting %)	Weight for height(Emaciation)
Great Tunis	98.10 (0.63)	93.25 (2.12)	96.21 (0.69)
North East	97.80 (0.82)	90.23 (2.7)	98.24 (0.40)
North West	95.55 (2.13)	86.94 (4.94)	98.15 (0.47)
Center east	98.26 (0.79)	94.15 (0.90)	97.22 (1.03)
Kasserine	98.15 (1.06)	86.17 (2.52)	97.41 (0.96)
Kairouan	96.23 (2.30)	86.88 (4.95)	98.28 (1.16)
Sidi Bouzid	95.74 (1.5)	86.80 (3.47)	97.02 (1.71)
South East	98.07 (0.42)	93.08 (2.16)	97.32 (1.21)
South West	95.02 (1.63)	86.25 (2.83)	97.28 (1.07)
Tunisia	97.43 (0.6)	89.66 (2.18)	97.79 (0.42)

Numbers in parenthesis are corresponding D-index values

The first indicator that measures both acute and chronic malnutrition (weight-for-age) is 97.43% meaning that 97.43% of children among all population of reference have the opportunity to be well nourished. The corresponding D-index (which measures inequality) implies that 0.6% of opportunities must be redistributed fairly to ensure equality of opportunity in terms of protection against malnutrition. Thus, associated HOI which is coverage penalized for inequality (C * (1-D)] is estimated to be 96.8%.

Concerning height for age which measures linear growth, we can see that 89.66% of Tunisian’s children have the opportunity to grow normally with a slow D-index of 2.18% and a HOI of 87.71%. Finally, the latest nutritional weight-for-height indicator (which measures emaciation) shows a coverage rate of 97.79%. That is, 97.79% of children in Tunisia have the opportunity to be sufficiently and efficiently nourished.

Despite the high level of anthropometric indicators throughout the country, there is a disparity between regions. Indeed, weight-for-age (which detects both acute and chronic malnutrition) is found to be low in inland areas compared to littoral regions. For example, in Sidi Bouzid, in the South West, in Kairoaun and in the North West, 95.74%; 95.03%; 96.23% and 95.55% are respectively found, while in district Tunis and in the Center East we find 98.10% and 98.26%, respectively.

Similarly, height for age which is a linear growth indicator and weight-for-age (the indicator of emaciation) are also low in western and inland regions (such as kairouan and sidi bouzid and middle west) than in regions in the east of the country (littoral) as shown in the Table 4 below, showing the regional coverage for 3 nutritional indicators.

Otherwise, Table 4 shows that anthropometric indicators vary according to socio-demographic and regional criteria in Tunisia. Despite good nutritional indices at the national level, it seems that there are many regional imbalances and disparities in access to these primary services. In this sense, it appears that children in the western, southwestern regions (with low coverage) are more susceptible to suffer from stunting, problems of emaciation and underweight (Malnutrition). For example, South west region presents the lowest rate of coverage against stunting problem (only 86.25% of children are protected) while the center east present the highest level of coverage (with more than 94.00%). Concerning dissimilarity at the same region, we note that children of the center east are more mo meaning that they have comparable chances to be covered against stunting (less than 1%). For children living in North West and Kairouan inequality between childhoods in terms of protection against nutritional problems is again remarkable (D-index = 4.95% for stunting problem in Kairouan). To give sense to our analysis and searching to quantify the contribution of circumstances variables in explaining inequality we are based on the Shapley decomposition and results are presented below:

Table 5 illustrates a Shapley decomposition result which consists at identifying sources of dissimilarity in terms of anthropometric services. From this table, it appears that the “household size” best explains both acute and chronic malnutrition of children followed by ‘head’s household age’. This result confirms our conclusions based on Table 3 such us this two variables are strongly significant in explaining malnutrition of Tunisian’s children. For stunting situation, we can see that the main determinant of delays in children growth is the family economic situation and head’s household education and that this finding is supported by the significance of these variables at the 5% threshold in Logit regression. Then, the number of child per household is an important factor explaining emaciation of early childhood in Tunisia. Furthermore, we note that the variables region is significant in explaining nutritional status of children meaning that people living in the west are favored than the rest of citizens (Table 10 Appendix).

**Table 5:** Shapely decomposition of regional nutritional disparities by circumstances

	Gender	Residence	Head’s household education	Wealth index	Household gender	Household size	Head’s Household age	Number of children per household	All regions
Weight for age (malnutrition)	0.79	2.44	10.42	15.33	5.40	37.27	23.57	4.73	35.26
Height for age (stunting)	0.16	25.49	11.71	43.58	8.01	4.76	2.55	3.71	22.31
Weight for height (Emaciation)	16.05	5.16	3.72	4.97	7.42	7.35	7.89	47.39	22.50

#### Discussions

Our results show that inequalities in terms of nutritional conditions are largely explained by economic indicators such us wealth index or number of children per household. These variables are different between eastern and western regions (Table 10 Appendix) which explains differences in terms of coverage and dissimilarity in access to basic nutritional services presented in Table 4.

In one hand, the western regions are of low demographic concentration compared to the coastal regions. On the other hand, the households living in these regions are mostly in rural areas which are characterized by a delicate financial situation and a low income (In some families no one have a permanent work). For example, the poorest family income represent 58.08% in Sidi Bouzid against only 10.06% in Center east (Table 10 Appendix). In addition to the lack of investment in these regions (compared to coastal regions which seduce investors), basic infrastructure and public health institutions are inexistent or under developed(for example access to potable water is 70.22% in district Tunisia but does not exceed 36% in Sidi Bouzid or 44.59% in Kairouan (Table 10 Appendix). Moreover theses regions are characterized by a low level of parents’ education reducing chance for child to receive appropriate vaccine and nutrition. For example, women who have not received any training account for roughly 33% in kairouan and sidi sidi bouzid while in the center it is not more than 7%.

All these conditions influence the environment in which the child is born and is obliged to survive in a difficult nutritional situation affecting its intellectual capacities and productive skills. In rural area 13.78% of children are exposed to growth problem against 7.84% in urban regions (Table 1). These results can be explained by inefficient intervention of public authorities to overcome social problems and reduce differences of inequality between regions. In developing countries, such as Tunisia, the state is in the center of economy and public sector still dominates. So, inequality in access to basic service is largely explained by absence of an efficient and equitable policy of income redistribution by public authorities on the basis of a fiscal policy driven by high rates against the rich and subsidies addressed to the poorest agents. Private sector is still underdeveloped or embryonic and its role of redistribution of profits is non-existent or negligible because of inappropriate institutional framework or absence of good governance. Regions that are characterized by problem of economic growth, high levels of poverty and lack of infrastructure are characterized by childhood opportunity inequalities, reduced feelings of Non-membership and criminal in adulthood. Many statistics on terrorism consider Tunisia as leader in terms of terrorism explaining this phenomenon by poverty, lack of social equity and unequal opportunities. These latter can be more serious in adulthood because of the differences in efforts which themselves depend on circumstances uncontrollable by agents.

In order to test robustness of our findings, we present significance of each variables using Logit model regression by region in the appendices (Table 16 Appendix). We mainly conclude that head’s household education, family income and head’s household age matters in disadvantaged areas but does not arise in more developed regions in explaining nutritional insufficiency. Results are largely similar to our main regressions and confirm our interpretations and conclusions.

### Access to health care services before, after, and during pregnancy

As mentioned above, the use of prenatal and postnatal care and during pregnancy are very important for the development of the child. So, similarly to our demarche in subsection 4.1 in the case of nutritional status of Tunisian childhood, we begin by presenting results of logit model in order to specify principal determinants of each healthy indicator.

#### Results

Table 6 shows the results of Logit model regression when we consider health indicator variables as dependant variables. The second column shows that coefficients associated to the variables residence, head’s household education, gender and age, household size, and numbers of children are statistically significant at the 10% threshold in explaining access to prenatal care during the full sample period. In 2012, residence and household’s age become insignificant but we can see that male children have less possible access to prenatal service(the coefficient of gender variable is statistically significant at conventional level). Concerning blood sample during the period 1982-2012, we note that access to this service is totally explained by the same determinants of prenatal services but no variables are significant in 2012. Finally, access to post natal care are largely explained by family income, number of children between 2 and 14 years and head’s household age for our two subsample in addition to insignificant role of residence and household size in 2012 compared to the full sample.

**Table 6:** Results of logit model (Health)

	Tunisia 1982-2012						Tunisia 2011-2012					
Endogenous variables	Prenatal care		Blood samples		Postnatal care		Prenatal care		Blood samples		Postnatal care	
Exogenous Variables	Coef	*P*-Value	Coef	*P*-Value	Coef	*P*-Value	Coef	*P*-Value	Coef	*P*-Value	Coef	*P*-Value
Gender	.074	0.344	.099	0.209	.011	0.901	−.907	0.056	.002	0.993	−.115	0.359
Residence	−.267	0.006	−.243	0.012	−.275	0.017	−.272	0.616	.125	0.711	−.149	0.337
H-h Education	−.250	0.057	−.296	0.025	−.122	0.457	1.28	0.021	.146	0.738	.209	0.360
Wealth index	−.008	0.931	.012	0.895	.192	0.097	.570	0.337	.457	0.193	.372	0.015
H-h gender	.389	0.039	.386	0.043	.368	0.122	1.35	0.068	.652	0.271	.045	0.888
Household size	.345	0.000	.333	0.000	.331	0.000	−.205	0.245	−.144	0.225	.083	0.197
Number of children(2-14)	−.750	0.000	−.741	0.000	−.676	0.000	−.423	0.071	−.214	0.160	−.161	0.043
H-h age	−.112	0.000	−.111	0.000	−.107	0.000	.037	0.122	.012	0.452	−.013	0.073
Constant	3.27	0.000	3.19	0.000	2.07	0.000	2.27	0.074	2.41	0.013	.368	0.462
Obs	4200		4200		4200		1059		1059		1059	
Prob > chi2	0.0000		0.0000		0.0000		0.0000		0.0059		0.0122	

Table 7 shows that at the national level, access to the prenatal services is seen to be very limited, with 24.66% of mothers in Tunisia received prenatal services during the period from 1982 until 2012. In other words, almost a quarter of Tunisian children have the opportunity to access to prenatal care services. Therefore, D-index (which measures inequality) is high meaning that 27.95% of Tunisian prenatal services are granted in an unequal manner and need to be redistributed equally to ensure equal opportunities (Corresponding HOI is small and does not exceed 17.77%). Similarly for the other indicators, it was found that 23.85% of mothers received blood samples to detect nutritional deficiencies in their offspring, and only 14.33% benefited from postnatal services such as midwifery or trained staff.

**Table 7:** Coverage rate of access to health indicators by regions (1982-2012)

Tunisia 1982-2012	Access to prenatal care %	Access to blood samples %	Access to postnatal care %
Great Tunis	20.98 (38.84)	20.66 (39.18)	12.40 (44.37)
North East	24.40 (33.32)	23.37(33.59)	17.23 (nn.29)
North West	21.51 (22.48)	20.54(21.74)	12.40 (30.19)
Center East	22.59(36.95)	21.54(36.40)	18.20 (39.24)
Kasserine	25.19(27.25)	23.91(29.18)	16.53(31.86)
Kairouan	32.32 (21.66)	31.78(21.79)	16.16 (24.21)
Sidi Bouzid	24.13(27.49)	22.70(26.04)	12.50 (31.51)
South East	28.81(25.10)	28.60(25.49)	12.07(18.19)
South West	25.42 (28.59)	24.69(28.23)	12.10(30.79)
Tunisia	24.66(27.95)	23.85(28.02)	14.33(30.76)

Numbers in parenthesis are corresponding D-index values

Despite the limited coverage rates in previous decades, the Tunisian Government has greatly improved its prenatal and postnatal services during the last few years. Table 8 shows that 97.82% and 94.61% of Tunisian childhood have access to prenatal care and blood sample, respectively, in 2012 with a small dissimilarity index (0.977%). But, the level of access to postnatal services remains low since half of the children do not have access to this service (only 56.84% have access to postnatal services).

**Table 8:** Coverage rate of access to health indicators by regions (2011-2012)

Tunis 2011-2012	Access to prenatal care %	Access to blood samples %	Access to postnatal care %
Coast regions	98.66 (.523)	96.00 (.834)	61.40(3.95)
Interior regions	96.99 (1.65)	93.24(2.06)	52.34(6.27)
Male	97.06(1.39)	94.67(1.60)	55.41(4.62)
Female	98.63(.693)	94.55 (1.20)	58.36(5.71)
Urban	98.50(.827)	96.00(.923)	58.06(3.73)
Rural	96.94 (1.18)	92.79(1.42)	55.24(6.95)
Nord	98.21(.710)	94.91(1.28)	61.83(5.52)
Center	98.10(.771)	94.56(1.46)	63.74(6.76)
South	93.97 (3.45)	94.32(2.30)	44.17(6.76)
Tunisia	97.82(.977)	94.61(1.32)	56.84(4.74)

Numbers in parenthesis are corresponding D-index values

Table 7 shows that there are important disparities between regions and socio-demographic neighborhoods in Tunisia during the period 1982-2012. This table shows that for access to prenatal services, most of the eastern regions of the country in addition to Kairoaun have higher coverage rate than the rest of the regions, ie children of these regions have most opportunity to access to these services compared to other regions. For example in Kairouan 32.32%, and in the South East 28.81% of child or (mother) received prenatal care (vaccinations), while in North west 21.51% of concerned population have the chance to receive the same services with a high dissimilarity index in eastern region (for example D-index in center east is 36.95% which is very high for a country in the Mediterranean basin) meaning that most of childhood have not received the same opportunities to benefit from this service. In 2012, access to prenatal is improved in all regions approaching 100% and disparities are reduced with a small advantage of cost regions compared to interior regions (and urban region are more covered by this service). If we decompose Tunisian area into three great zones, we feel that southern governorates are less favored in access to prenatal services (HOI = 93.97% even that D-index is small and do not exceed 4% (Table 8).

For the other indicators, regional disparities in access to post-natal services and blood sampling are discarded. Indeed, for blood sampling, Sidi Bouzid and the South West have the lowest coverage rates and they also remain for the postnatal indicator during the full sample period. For the last indicator (postnatal care), only the Central East and North East regions have the highest rate. In 2012, there are no great differences between male and female in access to blood sample and post natal services. But, coast and urban regions are more covered by these services than others zones especially southern and interior regions.

To identify exogenous variable that contributes more to differences of inequality we presented Shapley decomposition results (Table 9). The main finding is that the variable “head’s household age” is the most important to explain inequality of access to all health services during the last three decades. Surprisingly, this variable is the most significant in explaining discrepancy in terms of access to health services. Thus, an inequality grows over time and become very serious in adulthood or when agents become older. This reality can be, in part, explained by education level of the head’s household but may also be the consequence of an inappropriate health system that does not care for the elderly. Many households are not part of the health insurance system and spend most of their working lives in black jobs. This fragile labor situation, generally without social contributions, leads to retirement age without social security benefits. Head’s household age is again important in explaining access to post natal care but the variable “number of children (2-14)” prevails in 2012 in explaining opportunity’ inequality in access to prenatal care and blood samples. In addition, we remark that family income begins to become important determinant of health services access in lat years. These conclusions are largely supported by results obtained by logit model regression (Table 6).

**Table 9:** Decomposition of dissimilarity in access to health care services by circumstances

Tunisia: 1982-2012	Gender	Residence	Head’s household education	Wealth index	Household gender	Household size	Household age	Number of children per household	All regions
Prenatal care	.716	4.38	2.03	2.03	1.82	8.44	44.67	30.13	5.76
Blood Samples	1.02	3.88	1.85	1.66	1.74	8.72	44.33	30.39	.363
Postnatal Care	.221	3.16	2.86	.248	2.05	8.84	46.78	28.21	7.59
Tunisia: 2012									
Prenatal care	10.61	5.88	13.47	12.22	6.64	15.28	2.60	25.56	7.69
Blood Samples	.296	13.31	3.35	20.93	3.58	19.08	2.06	25.84	11.51
Postnatal Care	4.62	4.16	8.29	21.89	1.94	6.58	13.42	12.48	26.57

#### Discussions

In fact, mothers who need more health care before, during and after pregnancy are in areas of low demographic or rural concentration, especially in the western and Southern regions and in Sidi Bouzid as we have already seen. Despite the similar level of coverage in some cases, the qualification of the officers performing this service differs widely across regions (Table 15 Appendix). Coverage rates are smaller compared to others regions. In addition residence, household education and wealth income are statistically significant in explaining access to health services in many regions of the south and west which is not the case for eastern region (Table 17 Appendix). Moreover, the infrastructure in these interior governorates is almost not-existent; hence moving for diagnosis is difficult for too old mothers. Health information and advices for the mother during the pregnancy phase are considered as a lever for the future development of the babies. However, women living in these areas have low levels of education. As a result, the prevalence of diseases caused by lack of health care has been observed among children from the poorest households and the least educated and elderly mothers.

As a conclusion, families characterized by numerous children and older head’s household are more exposed to health problems in all the whole territory. In particular, the southern region are less favored in access to prenatal and postnatal care services in addition to the qualification of personnel ensuring this task. This fact can be explained by the absence of health schools and university hospitals in addition to specialized medicine in these regions.

## Conclusion and policy implications

Deficits and inequality early in life tend to accumulate and compound and lead to persistent shortfalls in human capital [32]. Based on a relatively few circumstances, which are entirely beyond of their control, this paper has shown that, Tunisian children face unequal opportunities to develop in terms of health, nutrition, cognitive, social, and emotional development. Likewise, we found that, parents’ education, wealth, age of household head and geographic factors as key factors determining child development outcomes.

Unequal provision of government services across different regions could contribute to geographic differences. Thus, it was recommended, among other things, that the government should, make periodic surveys on health status, on health care utilization, for financial reasons, Furthermore, to reduce financial constraint on access to care, through better targeting of the poor who should benefit from free medical assistance.

It was further recommended that efforts should be made by policymakers to help and encourage doctors to settle specially in disadvantaged region. Finally, and, on the institutional side: the policymakers should pursue new plan to reduce social and regional inequalities in access to health service in particular in rural areas.

As a final recommendation, Tunisian State must restructure the pension funds and provide free services to children whose heads of households are not members of the social funds. This policy can help reducing inequalities of opportunity in adult age and so reducing criminals and terrorism and enhances growth and development through increased productivity.

## Appendix

**Table 10:** Sample’s characteristics by regions (Nutrition)

Tunisia		Gender		Residence		Household’s education				Mather’s education				Annual family incomes(Economic quintile)					Number of Children (2-14) at Home		Housing property			Waetr access: potable water	
		Male	Female	Urbain	Rural	Primary and similar	Secondary and similar	Superior	nothingness	Primary and similar	Secondary and similar	Superior	nothingness	The poorest	second	medium	fourth	the richest	Less than 3	3 and mores	proprietor	location	Other	No access	access
Region	Total	1482 53.54	1286 46.46	1607 58.06	1161 41.94	1135 41.00	956 34.54	350 12.64	327 11.81	917 33.13	951 34.36	434 15.68	466 16.84	737 26.63	606 21.89	479 17.30	565 20.41	381 13.76	1990 71.89	778 28.11	1970 71.17	480 17.34	318 11.49	1227 44.33	1541 55.67
	District Tunis	181 50.84	175 49.16	326 91.57	30 8.43	117 32.87	147 41.29	72 20.22	20 5.62	91 25.56	153 42.98	96 26.97	16 4.49	11 3.09	57 16.01	75 21.07	95 26.69	118 33.15	290 81.46	66 18.53	174 48.88	114 32.02	68 19.10	106 29.78	250 70.22
	Nord Est	212 55.94	167 44.06	193 50.92	186 49.08	169 44.59	126 33.25	54 14.25	30 7.92	128 33.77	165 43.54	54 14.25	32 8.44	63 16.62	91 24.01	75 19.79	84 22.16	66 17.41	310 81.79	69 18.2	278 73.35	56 14.78	45 11.87	153 40.37	226 59.63
	Nord Ouest	170 58.42	121 41.58	134 46.05	157 53.95	106 36.43	86 29.55	33 11.34	66 22.68	96 32.99	84 28.87	43 14.78	68 23.37	80 27.49	76 26.12	67 23.02	41 14.09	27 9.28	217 74.57	74 25.43	207 71.13	57 19.59	27 9.28	108 37.11	183 62.89
	Centre Est	164 53.25	144 46.75	222 72.08	86 27.92	127 41.23	112 36.36	47 15.26	22 7.14	97 31.49	113 36.69	75 24.35	23 7.47	31 10.06	58 18.83	51 16.56	100 32.47	68 22.08	232 75.32	76 24.68	218 70.78	78 25.32	12 3.90	126 40.91	182 59.09
	Kasserine	144 51.06	138 48.94	94 33.33	188 66.67	164 58.16	68 24.11	10 3.55	40 14.18	122 43.26	67 23.76	15 5.32	78 27.66	123 43.62	92 32.62	35 12.41	25 8.87	7 2.48	174 61.70	108 38.30	179 63.48	29 10.28	74 26.24	103 36.52	179 63.48
	Kairouan	171 56.07	134 43.93	129 42.30	176 57.70	119 39.02	106 34.75	29 9.51	51 16.72	114 37.38	68 22.30	21 6.89	102 33.44	165 54.10	59 19.34	36 11.80	38 12.46	7 2.30	192 62.95	113 37.05	242 79.34	31 10.16	32 10.49	169 55.41	136 44.59
	Sidi Bouzid	137 54.80	113 45.20	79 31.60	171 68.40	94 37.60	77 30.80	31 12.40	48 19.20	70 28.00	67 26.80	30 12.00	83 33.20	147 58.80	41 16.40	26 10.40	21 8.40	15 6.00	162 64.80	88 35.2	198 79.20	28 11.20	24 9.60	160 64.00	90 36.00
	Sud Est	175 50.43	172 49.57	255 3.49	92 26.51	138 39.77	138 39.77	46 13.26	25 7.20	112 32.28	146 42.07	57 16.43	32 9.22	57 16.43	76 21.90	68 19.60	99 28.53	47 13.54	245 70.61	102 29.39	274 78.96	48 13.83	25 7.21	212 61.10	135 38.90
	Sud Ouest	128 51.20	122 48.80	175 70.00	75 30.00	101 40.40	96 38.40	28 11.20	25 10.00	87 34.80	88 35.20	43 17.20	32 12.80	60 24.00	56 22.40	46 18.40	62 24.80	26 10.40	168 67.20	82 32.8	200 80.00	39 15.60	11 4.40	90 36.00	160 64.00

**Table 11:** Sample’s characteristics by region (Health: 1982-2012)

Tunisia 1982-2012		Gender		Residence		Household’s education				Mather’s education				Annual family incomes(Economic quintile)					Number of Children (2-14) at Home		Housing property			Waetr access: potable water	
		Male	Female	Urbain	Rural	Primary and similar	Secondary and similar	Superior	nothingness	Primary and similar	Secondary and similar	Superior	nothingness	The poorest	second	medium	fourth	the richest	Less than 3	3 and mores	proprietor	location	Others	No access	access
Région	Total	2084 49.62	2116 50.38	2613 62.21	1587 37.79	1779 42.36	1380 32.86	443 10.55	598 14.24	645 36.05	538 30.07	201 11.24	405 22.64	1047 24.93	850 20.24	774 18.43	791 18.83	738 17.57	3272 77.90	928 22.1	3224 76.76	605 14.40	369 8.83	1741 41.45	2459 58.55
	District Tunis	292 46.42	337 53.58	583 92.69	46 7.31	222 35.29	240 38.16	117 18.60	50 7.95	93 35.63	106 40.61	47 18.01	15 5.75	18 2.86	98 15.58	125 19.87	136 21.62	252 40.06	534 84.90	95 15.1	374 59.46	165 26.23	90 14.31	183 29.09	446 70.91
	Nord Est	313 53.41	273 46.59	362 61.77	224 38.23	267 45.56	201 34.30	64 10.92	54 9.22	91 40.63	85 37.95	26 11.61	22 9.82	77 13.14	123 20.99	137 23.38	119 20.31	130 22.18	499 85.15	87 14.85	451 76.96	76 12.97	59 10.07	216 36.86	370 63.14
	Nord Ouest	257 49.81	259 50.19	232 44.96	284 55.04	218 42.2	132 25.5	43 8.33	123 23.84	59 27.96	47 22.27	24 11.37	81 38.39	171 33.14	118 22.87	112 21.71	71 13.76	44 8.53	421 81.59	95 18.41	400 77.52	69 13.37	47 9.11	197 38.18	319 61.82
	Centre Est	244 51.05	234 48.95	353 73.85	125 26.15	206 43.10	170 35.56	68 14.23	34 7.11	80 38.10	70 33.33	41 19.52	19 9.05	60 12.55	70 14.64	81 16.95	142 29.71	125 26.15	381 79.71	97 20.29	368 76.99	95 19.87	15 3.14	178 37.24	300 62.76
	Kasserine	164 41.73	229 58.27	151 38.42	242 61.58	195 49.62	105 26.72	16 4.07	77 19.59	68 39.53	38 22.09	6 3.49	60 34.88	172 43.77	107 27.23	50 12.72	38 9.67	26 6.62	267 67.94	126 32.06	282 71.76	38 9.67	73 18.58	141 35.88	252 64.12
	Kairouan	202 55.34	163 44.66	161 44.11	204 55.89	146 40.00	110 30.14	25 6.85	84 23.01	55 35.03	37 23.57	9 5.73	56 35.66	192 52.60	76 20.82	45 12.33	44 12.05	8 2.19	253 69.32	112 30.68	303 83.01	35 9.59	27 7.40	185 50.68	180 49.32
	Sidi Bouzid	174 50.00	174 50.00	114 32.76	234 67.24	144 41.38	101 29.02	35 10.06	68 19.54	42 25.61	34 20.73	14 8.54	74 45.12	199 57.18	59 16.95	38 10.92	31 8.91	21 6.03	239 68.68	109 31.32	296 85.06	35 10.06	17 4.89	216 62.07	132 37.93
	Sud Est	227 48.09	245 51.91	356 75.42	116 24.58	212 44.92	171 36.23	41 8.69	48 10.17	88 39.29	77 34.38	19 8.48	40 17.86	75 15.89	111 23.52	101 21.40	117 24.79	68 14.41	362 76.69	110 23.31	394 83.47	50 10.59	28 5.93	282 59.75	190 40.25
	Sud Ouest	211 51.09	202 48.91	301 72.88	112 27.12	169 40.92	150 36.32	34 8.23	60 14.53	69 41.57	44 26.51	15 9.04	38 22.89	83 20.10	88 21.31	85 20.58	93 22.52	64 15.50	316 76.51	97 23.49	356 86.20	42 10.17	15 3.63	143 34.62	270 65.38

**Table 12:** Sample’s characteristics by region (Health: 2011-2012).

Tunisia:2012		Gender		Residence		Household’s education				Mather’s education				Annual family incomes(Economic quintile)					Number of Children (2-14) at Home		Housing property			Waetr access: potable water	
		Male	Female	Urbain	Rural	Primary and similar	Secondary and similar	Superior	nothingness	Primary and similar	Secondary and similar	Superior	nothingness	The poorest	second	medium	fourth	the richest	Less than 3	3 and mores	proprietor	location	Others	No access	access
Région	Total	545 51.46	514 48.54	601 56.75	458 43.25	414 9.09	378 35.69	136 12.84	131 12.37	172 34.13	157 31.15	87 17.26	88 17.46	288 27.20	230 21.72	179 16.90	221 20.87	141 13.31	914 86.31	145 13.69	738 69.69	194 18.32	127 11.99	489 46.18	570 53.82
	District Tunis	61 45.19	74 54.81	125 92.59	10 7.41	43 31.85	52 38.52	31 22.96	9 6.67	16 25.81	25 40.32	19 30.65	2 3.23	6 4.44	19 14.07	26 19.26	37 27.41	47 34.81	131 97.04	4 2.96	57 42.22	53 39.26	25 18.52	40 29.63	95 70.37
	Nord Est	83 56.85	63 43.15	69 47.26	77 52.74	63 43.15	52 35.62	18 12.33	13 8.90	26 41.94	21 33.87	10 16.13	5 8.06	24 16.44	36 24.66	29 19.86	34 23.29	23 15.75	140 95.89	6 4.11	107 73.29	25 17.12	14 9.59	62 42.47	84 57.53
	Nord Ouest	55 49.11	57 50.89	46 41.07	66 58.93	39 34.82	33 29.46	11 9.82	29 25.89	14 27.45	9 17.65	13 25.49	15 29.41	40 35.71	27 24.11	21 18.75	15 13.39	9 8.04	99 88.39	13 2.68	84 75.00	17 15.18	11 9.82	44 39.29	68 60.71
	Centre Est	62 56.88	47 43.12	77 70.64	32 29.36	40 36.70	41 37.61	22 20.18	6 5.50	19 33.93	15 26.79	19 33.93	3 5.36	14 12.84	18 16.51	19 17.43	32 29.36	26 23.85	98 89.91	11 10.09	75 68.81	29 26.61	5 4.59	45 41.28	64 58.72
	Kasserine	45 44.12	57 55.88	32 31.37	70 68.63	52 50.98	27 26.47	5 4.90	18 17.65	20 41.67	14 29.17	2 4.17	12 25.00	39 38.24	37 36.27	13 12.75	11 10.78	2 1.96	83 81.37	19 18.63	60 58.82	13 12.75	29 28.43	37 36.27	65 63.73
	Kairouan	63 52.50	57 47.50	43 35.83	77 64.17	49 40.83	40 33.33	9 7.50	22 18.33	22 38.60	13 22.81	3 5.26	19 33.33	71 59.17	21 17.50	12 10.00	13 10.83	3 2.50	92 76.67	28 23.24	97 80.83	9 7.50	14 11.67	72 60.00	48 40.00
	Sidi Bouzid	58 61.70	36 38.30	28 29.79	66 70.21	35 37.23	30 31.91	13 13.83	16 17.02	15 28.30	16 30.19	6 11.32	16 30.19	47 50.00	20 21.28	11 11.70	11 11.70	5 5.32	73 77.66	21 22.34	76 80.85	10 10.64	8 8.51	62 65.96	32 34.04
	Sud Est	65 47.79	71 52.21	101 74.26	35 25.74	51 37.50	62 45.59	14 10.29	9 6.62	21 31.34	28 41.79	8 11.94	10 14.92	22 16.18	33 24.26	29 21.32	41 30.15	11 8.09	115 84.56	21 15.44	101 74.26	19 13.97	16 11.77	87 63.97	49 36.03
	Sud Ouest	53 50.48	52 49.52	80 76.19	25 23.81	42 40.00	41 39.05	13 12.38	9 8.57	19 39.58	16 33.33	7 14.58	6 12.50	25 23.81	19 18.10	19 18.10	27 25.71	15 14.29	83 79.05	22 20.95	81 77.14	19 18.10	5 4.76	40 38.10	65 61.90

The second value in Tables 10, 11 and 12 Appendix correspond to the percentage contribution in the corresponding sample

**Table 13:** Weight-for-age, and length –for –age, age in years and months

		Z-scores (weight in kg))								Z-scores (length in cm)							
		Weight-for-age for boys				Weight-for-age for girls				Length –for –age for boys,				Length –for –age for girls			
Year: month	Month	-3SD	-2SD	-1SD	Median	-3SD	-2SD	-1SD	Median	-3SD	-2SD	-1SD	Median	-3SD	-2SD	-1SD	Median
0:0	0	2.1	2.5	2.9	3.3	2.0	2.4	2.8	3.2	44.2	46.1	48.0	49.9	43.6	45.4	47.3	49.1
0:1	1	2.9	3.4	3.9	4.5	2.7	3.2	3.6	4.2	48.9	50.8	52.8	54.7	47.8	49.8	51.7	53.7
0:2	2	3.8	4.3	4.9	5.6	3.4	3.9	4.5	5.1	52.4	54.4	56.4	58.4	51.0	53.0	55.0	57.1
0:3	3	4.4	5.0	5.7	6.4	4.0	4.5	5.2	5.8	55.3	57.3	59.4	61.4	53.5	55.6	57.7	59.8
0:4	4	4.9	5.6	6.2	7.0	4.4	5.0	5.7	6.4	57.6	59.7	61.8	63.9	55.6	57.8	59.9	62.1
0:5	5	5.3	6.0	6.7	7.5	4.8	5.4	6.1	6.9	59.6	61.7	63.8	65.9	57.4	59.6	61.8	64.0
0:6	6	5.7	6.4	7.1	7.9	5.1	5.7	6.5	7.3	61.2	63.3	65.5	67.6	58.9	61.2	63.5	65.7
0:7	7	5.9	6.7	7.4	8.3	5.3	6.0	6.8	7.6	62.7	64.8	67.0	69.2	60.3	62.7	65.0	67.3
0:8	8	6.2	6.9	7.7	8.6	5.6	6.3	7.0	7.9	64.0	66.2	68.4	70.6	61.7	64.0	66.4	68.7
0:9	9	6.4	7.1	8.0	8.9	5.8	6.5	7.3	8.2	65.2	67.5	69.7	72.0	62.9	65.3	67.7	70.1
0:10	10	6.6	7.4	8.2	9.2	5.9	6.7	7.5	8.5	66.4	68.7	71.0	73.3	64.1	66.5	69.0	71.5
0:11	11	6.8	7.6	8.4	9.4	6.1	6.9	7.7	8.7	67.6	69.9	72.2	74.5	65.2	67.7	70.3	72.8
1:0	12	6.9	7.7	8.6	9.6	6.3	7.0	7.9	8.9	68.6	71.0	73.4	75.7	66.3	68.9	71.4	74.0
1:1	13	7.1	7.9	8.8	9.9	6.4	7.2	8.1	9.2	69.6	72.1	74.5	76.9	67.3	70.0	72.6	75.2
1:2	14	7.2	8.1	9.0	10.1	6.6	7.4	8.3	9.4	70.6	73.1	75.6	78.0	68.3	71.0	73.7	76.4
1:3	15	7.4	8.3	9.2	10.3	6.7	7.6	8.5	9.6	71.6	74.1	76.6	79.1	69.3	72.0	74.8	77.5
1:4	16	7.5	8.4	9.4	10.5	6.9	7.7	8.7	9.8	72.5	75.0	77.6	80.2	70.2	73.0	75.8	78.6
1:5	17	7.7	8.6	9.6	10.7	7.0	7.9	8.9	10.0	73.3	76.0	78.6	81.2	71.1	74.0	76.8	79.7
1:6	18	7.8	8.8	9.8	10.9	7.2	8.1	9.1	10.2	74.2	76.9	79.6	82.3	72.0	74.9	77.8	80.7
1:7	19	8.0	8.9	10.0	11.1	7.3	8.2	9.2	10.4	75.0	77.7	80.5	83.2	72.8	75.8	78.8	81.7
1:8	20	8.1	9.1	10.1	11.3	7.5	8.4	9.4	10.6	75.8	78.6	81.4	84.2	73.7	76.7	79.7	82.7
1:9	21	8.2	9.2	10.3	11.5	7.6	8.6	9.6	10.9	76.5	79.4	82.3	85.1	74.5	77.5	80.6	83.7
1:10	22	8.4	9.4	10.5	11.8	7.8	8.7	9.8	11.1	77.2	80.2	83.1	86.0	75.2	78.4	81.5	84.6
1:11	23	8.5	9.5	10.7	12.0	7.9	8.9	10.0	11.3	78.0	81.0	83.9	86.9	76.0	79.2	82.3	85.5
2:0	24	8.6	9.7	10.8	12.2	8.1	9.0	10.2	11.5	78.0	81.0	84.1	87.1	76.0	79.3	82.5	85.7
2:1	25	8.8	9.8	11.0	12.4	8.2	9.2	10.3	11.7	78.6	81.7	84.9	88.0	76.8	80.0	83.3	86.6
2:2	26	8.9	10.0	11.2	12.5	8.4	9.4	10.5	11.9	79.3	82.5	85.6	88.8	77.5	80.8	84.1	87.4
2:3	27	9.0	10.1	11.3	12.7	8.5	9.5	10.7	12.1	79.9	83.1	86.4	89.6	78.1	81.5	84.9	88.3
2:4	28	9.1	10.2	11.5	12.9	8.6	9.7	10.9	12.3	80.5	83.8	87.1	90.4	78.8	82.2	85.7	89.1
2:5	29	9.2	10.4	11.7	13.1	8.8	9.8	11.1	12.5	81.1	84.5	87.8	91.2	79.5	82.9	86.4	89.9
2:6	30	9.4	10.5	11.8	13.3	8.9	10.0	11.2	12.7	81.7	85.1	88.5	91.9	80.1	83.6	87.1	90.7
2:7	31	9.5	10.7	12.0	13.5	9.0	10.1	11.4	12.9	82.3	85.7	89.2	92.7	80.7	84.3	87.9	91.4
2:8	32	9.6	10.8	12.1	13.7	9.1	10.3	11.6	13.1	82.8	86.4	89.9	93.4	81.3	84.9	88.6	92.2
2:9	33	9.7	10.9	12.3	13.8	9.3	10.4	11.7	13.3	83.4	86.9	90.5	94.1	81.9	85.6	89.3	92.9
2:10	34	9.8	11.0	12.4	14.0	9.4	10.5	11.9	13.5	83.9	87.5	91.1	94.8	82.5	86.2	89.9	93.6
2:11	35	9.9	11.2	12.6	14.2	9.5	10.7	12.0	13.7	84.4	88.1	91.8	95.4	83.1	86.8	90.6	94.4
3:0	36	10.0	11.3	12.7	14.3	9.6	10.8	12.2	13.9	85.0	88.7	92.4	96.1	83.6	87.4	91.2	95.1
3:1	37	10.1	11.4	12.9	14.5	9.7	10.9	12.4	14.0	85.5	89.2	93.0	96.7	84.2	88.0	91.9	95.7
3:2	38	10.2	11.5	13.0	14.7	9.8	11.1	12.5	14.2	86.0	89.8	93.6	97.4	84.7	88.6	92.5	96.4
3:3	39	10.3	11.6	13.1	14.8	9.9	11.2	12.7	14.4	86.5	90.3	94.2	98.0	85.3	89.2	93.1	97.1
3:4	40	10.4	11.8	13.3	15.0	10.1	11.3	12.8	14.6	87.0	90.9	94.7	98.6	85.8	89.8	93.8	97.7
3:5	41	10.5	11.9	13.4	15.2	10.2	11.5	13.0	14.8	87.5	91.4	95.3	99.2	86.3	90.4	94.4	98.4
3:6	42	10.6	12.0	13.6	15.3	10.3	11.6	13.1	15.0	88.0	91.9	95.9	99.9	86.8	90.9	95.0	99.0
3:7	43	10.7	12.1	13.7	15.5	10.4	11.7	13.3	15.2	88.4	92.4	96.4	100.4	87.4	91.5	95.6	99.7
3:8	44	10.8	12.2	13.8	15.7	10.5	11.8	13.4	15.3	88.9	93.0	97.0	101.0	87.9	92.0	96.2	100.3
3:9	45	10.9	12.4	14.0	15.8	10.6	12.0	13.6	15.5	89.4	93.5	97.5	101.6	88.4	92.5	96.7	100.9
3:10	46	11.0	12.5	14.1	16.0	10.7	12.1	13.7	15.7	89.8	94.0	98.1	102.2	88.9	93.1	97.3	101.5
3:11	47	11.1	12.6	14.3	16.2	10.8	12.2	13.9	15.9	90.3	94.4	98.6	102.8	89.3	93.6	97.9	102.1
4:0	48	11.2	12.7	14.4	16.3	10.9	12.3	14.0	16.1	90.7	94.9	99.1	103.3	89.8	94.1	98.4	102.7
4:1	49	11.3	12.8	14.5	16.5	11.0	12.4	14.2	16.3	91.2	95.4	99.7	103.9	90.3	94.6	99.0	103.3
4:2	50	11.4	12.9	14.7	16.7	11.1	12.6	14.3	16.4	91.6	95.9	100.2	104.4	90.7	95.1	99.5	103.9
4:3	51	11.5	13.1	14.8	16.8	11.2	12.7	14.5	16.6	92.1	96.4	100.7	105.0	91.2	95.6	100.1	104.5
4:4	52	11.6	13.2	15.0	17.0	11.3	12.8	14.6	16.8	92.5	96.9	101.2	105.6	91.7	96.1	100.6	105.0
4:5	53	11.7	13.3	15.1	17.2	11.4	12.9	14.8	17.0	93.0	97.4	101.7	106.1	92.1	96.6	101.1	105.6
4:6	54	11.8	13.4	15.2	17.3	11.5	13.0	14.9	17.2	93.4	97.8	102.3	106.7	92.6	97.1	101.6	106.2
4:7	55	11.9	13.5	15.4	17.5	11.6	13.2	15.1	17.3	93.9	98.3	102.8	107.2	93.0	97.6	102.2	106.7
4:8	56	12.0	13.6	15.5	17.7	11.7	13.3	15.2	17.5	94.3	98.8	103.3	107.8	93.4	98.1	102.7	107.3
4:9	57	12.1	13.7	15.6	17.8	11.8	13.4	15.3	17.7	94.7	99.3	103.8	108.3	93.9	98.5	103.2	107.8
4:10	58	12.2	13.8	15.8	18.0	11.9	13.5	15.5	17.9	95.2	99.7	104.3	108.9	94.3	99.0	103.7	108.4
4:11	59	12.3	14.0	15.9	18.2	12.0	13.6	15.6	18.0	95.6	100.2	104.8	109.4	94.7	99.5	104.2	108.9
5:12	60	12.4	14.1	16.0	18.3	12.1	13.7	15.8	18.2	96.1	100.7	105.3	110.0	95.2	99.9	104.7	109.4

**Table 14:** Weight-for-length standards

	Z-scores (weight in kg)							
	Weight-for-length for boys				Weight-for-length for Girls			
Length (cm)	-3SD	-2SD	-1SD	Median	-3SD	-2SD	-1SD	Median
45.0	1.9	2.0	2.2	2.4	1.9	2.1	2.3	2.5
45.5	1.9	2.1	2.3	2.5	2.0	2.1	2.3	2.5
46.0	2.0	2.2	2.4	2.6	2.0	2.2	2.4	2.6
46.5	2.1	2.3	2.5	2.7	2.1	2.3	2.5	2.7
47.0	2.1	2.3	2.5	2.8	2.2	2.4	2.6	2.8
47.5	2.2	2.4	2.6	2.9	2.2	2.4	2.6	2.9
48.0	2.3	2.5	2.7	2.9	2.3	2.5	2.7	3.0
48.5	2.3	2.6	2.8	3.0	2.4	2.6	2.8	3.1
49.0	2.4	2.6	2.9	3.1	2.4	2.6	2.9	3.2
49.5	2.5	2.7	3.0	3.2	2.5	2.7	3.0	3.3
50.0	2.6	2.8	3.0	3.3	2.6	2.8	3.1	3.4
50.5	2.7	2.9	3.1	3.4	2.7	2.9	3.2	3.5
51.0	2.7	3.0	3.2	3.5	2.8	3.0	3.3	3.6
51.5	2.8	3.1	3.3	3.6	2.8	3.1	3.4	3.7
52.0	2.9	3.2	3.5	3.8	2.9	3.2	3.5	3.8
52.5	3.0	3.3	3.6	3.9	3.0	3.3	3.6	3.9
53.0	3.1	3.4	3.7	4.0	3.1	3.4	3.7	4.0
53.5	3.2	3.5	3.8	4.1	3.2	3.5	3.8	4.2
54.0	3.3	3.6	3.9	4.3	3.3	3.6	3.9	4.3
54.5	3.4	3.7	4.0	4.4	3.4	3.7	4.0	4.4
55.0	3.6	3.8	4.2	4.5	3.5	3.8	4.2	4.5
55.5	3.7	4.0	4.3	4.7	3.6	3.9	4.3	4.7
56.0	3.8	4.1	4.4	4.8	3.7	4.0	4.4	4.8
56.5	3.9	4.2	4.6	5.0	3.8	4.1	4.5	5.0
57.0	4.0	4.3	4.7	5.1	3.9	4.3	4.6	5.1
57.5	4.1	4.5	4.9	5.3	4.0	4.4	4.8	5.2
58.0	4.3	4.6	5.0	5.4	4.1	4.5	4.9	5.4
58.5	4.4	4.7	5.1	5.6	4.2	4.6	5.0	5.5
59.0	4.5	4.8	5.3	5.7	4.3	4.7	5.1	5.6
59.5	4.6	5.0	5.4	5.9	4.4	4.8	5.3	5.7
60.0	4.7	5.1	5.5	6.0	4.5	4.9	5.4	5.9
60.5	4.8	5.2	5.6	6.1	4.6	5.0	5.5	6.0
61.0	4.9	5.3	5.8	6.3	4.7	5.1	5.6	6.1
61.5	5.0	5.4	5.9	6.4	4.8	5.2	5.7	6.3
62.0	5.1	5.6	6.0	6.5	4.9	5.3	5.8	6.4
62.5	5.2	5.7	6.1	6.7	5.0	5.4	5.9	6.5
63.0	5.3	5.8	6.2	6.8	5.1	5.5	6.0	6.6
63.5	5.4	5.9	6.4	6.9	5.2	5.6	6.2	6.7
64.0	5.5	6.0	6.5	7.0	5.3	5.7	6.3	6.9
64.5	5.6	6.1	6.6	7.1	5.4	5.8	6.4	7.0
65.0	5.7	6.2	6.7	7.3	5.5	5.9	6.5	7.1
65.5	5.8	6.3	6.8	7.4	5.5	6.0	6.6	7.2
66.0	5.9	6.4	6.9	7.5	5.6	6.1	6.7	7.3
66.5	6.0	6.5	7.0	7.6	5.7	6.2	6.8	7.4
67.0	6.1	6.6	7.1	7.7	5.8	6.3	6.9	7.5
67.5	6.2	6.7	7.2	7.9	5.9	6.4	7.0	7.6
68.0	6.3	6.8	7.3	8.0	6.0	6.5	7.1	7.7
68.5	6.4	6.9	7.5	8.1	6.1	6.6	7.2	7.9
69.0	6.5	7.0	7.6	8.2	6.1	6.7	7.3	8.0
69.5	6.6	7.1	7.7	8.3	6.2	6.8	7.4	8.1
70.0	6.6	7.2	7.8	8.4	6.3	6.9	7.5	8.2
70.5	6.7	7.3	7.9	8.5	6.4	6.9	7.6	8.3
71.0	6.8	7.4	8.0	8.6	6.5	7.0	7.7	8.4
71.5	6.9	7.5	8.1	8.8	6.5	7.1	7.7	8.5
72.0	7.0	7.6	8.2	8.9	6.6	7.2	7.8	8.6
72.5	7.1	7.6	8.3	9.0	6.7	7.3	7.9	8.7
73.0	7.2	7.7	8.4	9.1	6.8	7.4	8.0	8.8
73.5	7.2	7.8	8.5	9.2	6.9	7.4	8.1	8.9
74.0	7.3	7.9	8.6	9.3	6.9	7.5	8.2	9.0
74.5	7.4	8.0	8.7	9.4	7.0	7.6	8.3	9.1
75.0	7.5	8.1	8.8	9.5	7.1	7.7	8.4	9.1
75.5	7.6	8.2	8.8	9.6	7.1	7.8	8.5	9.2
76.0	7.6	8.3	8.9	9.7	7.2	7.8	8.5	9.3
76.5	7.7	8.3	9.0	9.8	7.3	7.9	8.6	9.4
77.0	7.8	8.4	9.1	9.9	7.4	8.0	8.7	9.5
77.5	7.9	8.5	9.2	10.0	7.4	8.1	8.8	9.6
78.0	7.9	8.6	9.3	10.1	7.5	8.2	8.9	9.7
78.5	8.0	8.7	9.4	10.2	7.6	8.2	9.0	9.8
79.0	8.1	8.7	9.5	10.3	7.7	8.3	9.1	9.9
79.5	8.2	8.8	9.5	10.4	7.7	8.4	9.1	10.0
80.0	8.2	8.9	9.6	10.4	7.8	8.5	9.2	10.1
80.5	8.3	9.0	9.7	10.5	7.9	8.6	9.3	10.2
81.0	8.4	9.1	9.8	10.6	8.0	8.7	9.4	10.3
81.5	8.5	9.1	9.9	10.7	8.1	8.8	9.5	10.4
82.0	8.5	9.2	10.0	10.8	8.1	8.8	9.6	10.5
82.5	8.6	9.3	10.1	10.9	8.2	8.9	9.7	10.6
83.0	8.7	9.4	10.2	11.0	8.3	9.0	9.8	10.7
83.5	8.8	9.5	10.3	11.2	8.4	9.1	9.9	10.9
84.0	8.9	9.6	10.4	11.3	8.5	9.2	10.1	11.0
84.5	9.0	9.7	10.5	11.4	8.6	9.3	10.2	11.1
85.0	9.1	9.8	10.6	11.5	8.7	9.4	10.3	11.2
85.5	9.2	9.9	10.7	11.6	8.8	9.5	10.4	11.3
86.0	9.3	10.0	10.8	11.7	8.9	9.7	10.5	11.5
86.5	9.4	10.1	11.0	11.9	9.0	9.8	10.6	11.6
87.0	9.5	10.2	11.1	12.0	9.1	9.9	10.7	11.7
87.5	9.6	10.4	11.2	12.1	9.2	10.0	10.9	11.8
88.0	9.7	10.5	11.3	12.2	9.3	10.1	11.0	12.0
88.5	9.8	10.6	11.4	12.4	9.4	10.2	11.1	12.1
89.0	9.9	10.7	11.5	12.5	9.5	10.3	11.2	12.2
89.5	10.0	10.8	11.6	12.6	9.6	10.4	11.3	12.3
90.0	10.1	10.9	11.8	12.7	9.7	10.5	11.4	12.5
90.5	10.2	11.0	11.9	12.8	9.8	10.6	11.5	12.6
91.0	10.3	11.1	12.0	13.0	9.9	10.7	11.7	12.7
91.5	10.4	11.2	12.1	13.1	10.0	10.8	11.8	12.8
92.0	10.5	11.3	12.2	13.2	10.1	10.9	11.9	13.0
92.5	10.6	11.4	12.3	13.3	10.1	11.0	12.0	13.1
93.0	10.7	11.5	12.4	13.4	10.2	11.1	12.1	13.2
93.5	10.7	11.6	12.5	13.5	10.3	11.2	12.2	13.3
94.0	10.8	11.7	12.6	13.7	10.4	11.3	12.3	13.5
94.5	10.9	11.8	12.7	13.8	10.5	11.4	12.4	13.6
95.0	11.0	11.9	12.8	13.9	10.6	11.5	12.6	13.7
95.5	11.1	12.0	12.9	14.0	10.7	11.6	12.7	13.8
96.0	11.2	12.1	13.1	14.1	10.8	11.7	12.8	14.0
96.5	11.3	12.2	13.2	14.3	10.9	11.8	12.9	14.1
97.0	11.4	12.3	13.3	14.4	11.0	12.0	13.0	14.2
97.5	11.5	12.4	13.4	14.5	11.1	12.1	13.1	14.4
98.0	11.6	12.5	13.5	14.6	11.2	12.2	13.3	14.5
98.5	11.7	12.6	13.6	14.8	11.3	12.3	13.4	14.6
99.0	11.8	12.7	13.7	14.9	11.4	12.4	13.5	14.8
99.5	11.9	12.8	13.9	15.0	11.5	12.5	13.6	14.9
100.0	12.0	12.9	14.0	15.2	11.6	12.6	13.7	15.0
100.5	12.1	13.0	14.1	15.3	11.7	12.7	13.9	15.2
101.0	12.2	13.2	14.2	15.4	11.8	12.8	14.0	15.3
101.5	12.3	13.3	14.4	15.6	11.9	13.0	14.1	15.5
102.0	12.4	13.4	14.5	15.7	12.0	13.1	14.3	15.6
102.5	12.5	13.5	14.6	15.9	12.1	13.2	14.4	15.8
103.0	12.6	13.6	14.8	16.0	12.3	13.3	14.5	15.9
103.5	12.7	13.7	14.9	16.2	12.4	13.5	14.7	16.1
104.0	12.8	13.9	15.0	16.3	12.5	13.6	14.8	16.2
104.5	12.9	14.0	15.2	16.5	12.6	13.7	15.0	16.4
105.0	13.0	14.1	15.3	16.6	12.7	13.8	15.1	16.5
105.5	13.2	14.2	15.4	16.8	12.8	14.0	15.3	16.7
106.0	13.3	14.4	15.6	16.9	13.0	14.1	15.4	16.9
106.5	13.4	14.5	15.7	17.1	13.1	14.3	15.6	17.1
107.0	13.5	14.6	15.9	17.3	13.2	14.4	15.7	17.2
107.5	13.6	14.7	16.0	17.4	13.3	14.5	15.9	17.4
108.0	13.7	14.9	16.2	17.6	13.5	14.7	16.0	17.6
108.5	13.8	15.0	16.3	17.8	13.6	14.8	16.2	17.8
109.0	14.0	15.1	16.5	17.9	13.7	15.0	16.4	18.0
109.5	14.1	15.3	16.6	18.1	13.9	15.1	16.5	18.1
110.0	14.2	15.4	16.8	18.3	14.0	15.3	16.7	18.3

**Table 15:** Prenatal care coverage Percentage distribution of women aged 15-49 who gave birth in the two years preceding the survey by antenatal care staff, Tunisia, 2011-2012

Tunisia 2011-2012			Doctor	Nurse/Midwife	Auxiliary midwife	Traditional accoucheuse	No prenatal care received	Any staff
	Total	1059 100.00	837 79.03	18 1.69	471 44.47	1 0.09	23 2.17	1036 97.83
Gender	Male	545 51.46	424 77.65	7 1.28	244 44.77	1 0.18	16 2.94	529 97.06
	Female	514 48.54	413 80.35	11 2.14	227 44.16	0 0.00	7 1.36	507 98.64
Residence	Urbain	601 56.75	497 82.69	11 1.83	244 40.59	1 0.16	9 1.50	592 98.50
	Rural	458 43.25	340 74.23	7 1.52	227 49.56	0 0.00	14 3.06	444 96.94
Region	District Tunis	135 12.75	123 91.11	0 0.00	22 16.29	0 0.00	3 2.22	132 97.78
	Nord Est	146 13.79	130 89.04	6 4.10	55 37.67	0 0.00	3 2.05	143 97.95
	Nord Ouest	112 10.58	86 76.78	1 0.89	65 58.03	1 0.89	1 0.89	111 99.11
	Centre Est	109 10.29	102 93.57	1 0.91	35 32.11	0 0.00	1 0.92	108 99.08
	Kasserine	102 9.63	77 75.49	1 0.98	54 52.94	0 0.00	3 2.94	99 97.06
	Kairouan	120 11.33	81 67.50	0 0.00	57 47.50	0 0.00	2 1.67	118 98.33
	Sidi Bouzid	94 8.88	72 76.59	1 1.06	29 30.85	0 0.00	10 10.64	84 89.36
	South East	136 12.84	94 69.11	3 2.20	79 58.08	0 0.00	0 0.00	136 100.00
	South west	105 9.92	72 68.57	5 4.76	75 71.42	0 0.00	0 0.00	105 100.00
Mather’s education	Nothingness	88 17.46	59 67.04	0 0.00	42 47.72	1 1.13	2 2.33	84 97.67
	Primary and similar	172 34.13	130 75.58	1 0.58	82 47.67	0 0.00	3 1.74	169 98.26
	Secondary and similar	157 31.15	125 79.61	4 2.54	68 43.31	0 0.00	4 2.55	153 97.45
	Superior	87 17.26	81 93.10	2 2.29	23 27.38	0 0.00	0 0.00	87 100.00
	No reponse	555 52.40	442 79.63	11 2.41	256 46.12	0 0.00	12 2.16	543 97.84
Annual family incomes (Economic quintile)	The poorest	288 27.20	171 59.37	3 1.04	151 52.43	1 0.34	15 5.21	273 94.79
	Second	230 21.72	180 78.26	5 2.17	123 53.47	0 0.00	2 0.87	228 99.13
	Medium	179 16.90	144 80.44	2 1.11	87 48.60	0 0.00	5 2.79	174 97.21
	Fourth	221 20.87	204 92.30	5 2.26	80 36.19	0 0.00	1 0.45	220 99.55
	The richest	141 13.31	138 97.87	3 2.12	30 21.27	0 0.00	0 0.00	141 100.00

The second value in the table corresponds to the percentage contribution in the corresponding sample

**Table 16:** Logit model regression by regions (Nutrition conditions)

2012	Nutrition: Weight for Age									Nutrition: Height for Age									Nutrition: Weight for height								
Regions	District Tunis	Nord Est	N ord Ouest	Centre Est	Kasserine	Kairouan	Sidi Bouzid	South East	South west	District Tunis	Nord Est	Nord Ouest	Centre Est	Kasserine	Kairouan	Sidi Bouzid	South East	South west	District Tunis	Nord Est	Nord Ouest	Centre Est	Kasserine	Kairouan	Sidi Bouzid	South East	South west
Gender		x	x																								
Residence																											
H-H Education				x	x	x						x			x	x										x	
Household income											x				x	x											
H-H gender																	x										
Household size				x		x			x			x										x		x			
Number of children (2-14)									x	x														x			
H-H age					x	x						x			x										x		

x indicates statistical significance at the 10% threshold level.

**Table 17:** Logit model regression by regions (Health care access)

1982-2012	Health: Prenatal care									Health: Blood samples									Health: Postnatal care								
Regions	District Tunis	Nord Est	Nord Ouest	Centre Est	Kasserine	Kairouan	Sidi Bouzid	South East	South west	District Tunis	Nord Est	N ord Ouest	Centre Est	Kasserine	Kairouan	Sidi Bouzid	South East	South west	District Tunis	Nord Est	Nord Ouest	Centre Est	Kasserine	Kairouan	Sidi Bouzid	South East	South west
GENDER							x	x								x									x		
Residence						x					x				x				x								
H-H Education												x														x	
Household income							x																x				
H-H gender																	x										
Household size	x	x	x	x	x	x	x	x	x	x	x	x	x	x	x	x	x	x	x	x	x	x	x	x		x	
Number of children (2-14)	x	x	x	x	x	x	x	x	x	x	x	x	x	x	x	x	x	x	x	x	x	x	x	x	x	x	
H-H age	x	x	x	x	x	x	x	x	x	x	x	x	x	x	x	x	x	x	x	x	x	x	x	x	x	x	

x indicates statistical significance at the 10% threshold level.

## Abbreviations

HOI: Human opportunity index
INS: National institute of statistics
MICS: Multiple indicator cluster surveys
UNESCO: United Nations, Education, scientific and Cultural Organization
UNFPA: United Nations population fund
UNICEF: United Nations Children’s Emergency Fund
WHO: world Health Organization

## Footnotes

1 In 2006, WHO published growth standards for weight and height to replace the 1977 National Center of Health Statistics (NCHS).

2 Chantreuil and Trannoy [28] and Sastre et Trannoy [29] applied Shapley decomposition methodology to explain only income inequality but Shorrocks [13] has shown that such a decomposition could be applied to any function.

3 Each of the three indicators of nutritional status can be expressed in units of standard deviations (reduced deviation) from the median of the reference population. The reference population used in this paper is based on the WHO growth standards. http://www.who.int/childgrowth/standards/second_set/technical_report_2.pdf. (Table A.3; A.4 and A.5 in appendix)

4 When *P*-Value is less than 5% we can reject the null hypothesis meaning that the coefficient is not significant. So, we accept alternative hypothesis which means that the variable is statistically significant in explaining dependent variable.
